# Flow cytometric analysis of hepatopancreatic cells from *Armadillidium vulgare* highlights terrestrial isopods as efficient environmental bioindicators in ex vivo settings

**DOI:** 10.1007/s11356-023-31375-x

**Published:** 2024-01-09

**Authors:** Giovanna Panza, Mariele Montanari, Daniele Lopez, Sabrina Burattini, Caterina Ciacci, Piermarco Paci Fumelli, Giovanni Pasini, Vieri Fusi, Luca Giorgi, Francesco Grandoni, Stefano Papa, Riccardo Santolini, Barbara Canonico

**Affiliations:** 1 Department of Biomolecular Sciences (DISB), University of Urbino Carlo Bo, 61029 Urbino, Italy; 2 Department of Pure and Applied Sciences (DiSPeA), University of Urbino Carlo Bo, 61029 Urbino, Italy; 3 Centro Ricerche Ecologiche E Naturalistiche (CREN), Soc. Coop., 47922 Rimini, Italy; 4 Centro Di Ricerca Zootecnia E Acquacoltura (Research Centre for Animal Production and Aquaculture), CREA — Consiglio per la Ricerca in Agricoltura e l’Analisi dell’Economia Agraria, Monterotondo, Rome Italy; 5 Department of Humanistic Studies (DISTUM), University of Urbino Carlo Bo, 61029 Urbino, Italy

**Keywords:** Terrestrial isopods, Environmental bioindicators, Biologic indicators, Hepatopancreas, Flow cytometry, Ecotoxicology, Environmental diagnosis

## Abstract

Several studies have reported the high bioindication capacity of Isopoda (Crustacea, Oniscidea), which is related to their important ability to accumulate contaminants, usefulness in soil ecotoxicology and bioindication activities. Any change in the isopod population, diversity and life cycle can indicate relevant pollution levels. The analysis of target tissues, such as the hepatopancreas, is another emerging approach (from a cytologic/histological level) to detect contaminant accumulation from different sources. In this study, tissue disaggregation procedures were optimised in the hepatopancreas, and flow cytometry (FC) was applied to detect cell viability and several cell functions. After disaggregation, two hepatopancreatic cell types, small (S) and big (B), were still recognisable: they differed in morphology and behaviour. The analyses were conducted for the first time on isopods from sites under different conditions of ecological disturbance through cytometric re-interpretation of ecological-environmental parameters. Significant differences in cell functional parameters were found, highlighting that isopod hepatopancreatic cells can be efficiently analysed by FC and represent standardisable, early biological indicators, tracing environmental-induced stress through cytologic/histologic analyses.

**Supplementary Information:**

The online version contains supplementary material available at 10.1007/s11356-023-31375-x.

## Introduction

Ecosystems, both natural and anthropic, are complex systems in which it is difficult to identify and analyse all the environmental factors that characterise them and regulate the balance and development of various organisms.

Considering that certain species/biocenoses can provide information on a site’s environmental characteristics, it simplifies the investigation’s complexity. Therefore, bioindicators become important tools for ecological investigation, facilitating the analysis and description of complex ecosystem settings. Terrestrial isopods change to are useful bioindicators for monitoring the environmental quality. Nevertheless, they are primarily employed as an important component of biodiversity in various natural and agricultural ecosystems (Solomou et al. 2019).

### Taxonomy

The order Isopoda, part of the phylum Arthropoda and subphylum Crustacea, is among the richest in terms of species and most biodiverse in relation to the diversity of their life and the variety of environments they frequently and counter (Dimitriou et al. 2019). They are the only group of crustaceans that has been able to colonise land massively (van Gestel et al. 2018).

In this study, we analysed *Armadillidium vulgare* (Latreille, 1804), a terrestrial species of *Armadillidium* (Crustacea, Isopoda, Oniscidea).

### Distribution

The original distribution of *Armadillidium vulgare* is throughout Europe, especially in the Mediterranean basin, but nowadays, it is a cosmopolitan species (Suzuki and Futami 2018).

European studies show a gradual decrease in the species richness of Armadillidiidae towards the north, which would also explain the fact that the highest isopod richness occurs in the Mediterranean area (Sfenthourakis and Hornung 2018).

### Bioindication features

An indicator is an organism or biological system used to assess a change, generally degenerative, in the quality of the environment, regardless of its level of organisation and use (Chowdhury et al. 2023).

Generally, a bioindicator is an organism or a biological system capable of providing information on one or more ecological phenomena in a given environment based on its presence/absence, abundance or changes in its biological, morphological and ecological status. Thus, they can be used for the recognition and qualitative-quantitative determination of environmental factors of anthropogenic origin (Chowdhury et al. 2023; Markert 2007).

In addition, the characteristics of a good indicator include providing an early warning of change (Chowdhury et al. 2023). The use of bioindicators plays a significant role in numerous fields, including environmental quality assessment, climate and land-use change surveys, analysis of the degree of anthropisation of a site, biodiversity analysis, land-use management and planning and environmental conservation and restoration (information on the loss, rarefaction and disappearance of certain environments) and ecological networks and corridors.

Every level of biological organisation can be used as a bioindicator, including communities, species, organisms, organs, tissues and cells. Bioindicators are frequently used as markers for biological parameters (Chowdhury et al. 2023).

### Bioindication and hepatopancreas

Cells can be studied as biomarkers able to measure the quality of the environment through definite biochemical, genetic, morphological or physiological changes. Variations in the biological cycle and accumulation of toxic substances in the tissues of organisms can be measured as a response to exposure to a stressor, usually polluting compounds.

Thus, the more specific the biomarker, the more unequivocally it will be possible to trigger of the contamination and, in rare cases, even to a specific contaminant.

Many studies have reported the high capacity of bioindication of Isopoda, especially its capacity to accumulate contaminants (Dallinger et al. 1992; Pastorino et al. 2021; van Gestel et al. 2018). They are also used as useful bioindicators of environmental contamination by heavy elements, which they uptake and accumulate (Odendaal and Reinecke 2004a, 2004b; Paoletti and Hassall 1999; Vijver et al. 2006; Witzel 1998).

In particular, they are capable of accumulating copper and cadmium (Hopkin and Martin 1982; Prosi and Dallinger 1988; van Gestel et al. 2018), zinc and lead, as found in lysosomes in the hepatopancreas (Drobne and Štrus 1996; Sommaggio et al. 2018), as well as storage for agricultural pesticides (e.g. fungicides such as hexachlorobenzene) (Kampe and Schlechtriem 2016; Mazzei et al. 2014; van Gestel et al. 2018). They can also modify the composition of various chemicals by increasing their solubility and the ability to be excreted (e.g. polycyclic aromatic hydrocarbons eliminated in less than a day) (van Gestel 2012). In addition, some studies have been conducted and measured mercury levels in relation to distance from highways and industrial sites (Chow 1970; Lerche and Breckle 1974; Smith 1972; Wieser et al. 1976).

Isopods are relevant models in soil ecotoxicology in laboratory toxicity tests, in field monitoring and bioindication studies (van Gestel 2012; van Gestel et al. 2018). Isopods are terrestrial invertebrates that provide information on soil contamination levels (Pastorino et al. 2021).

**Fig. 1:**
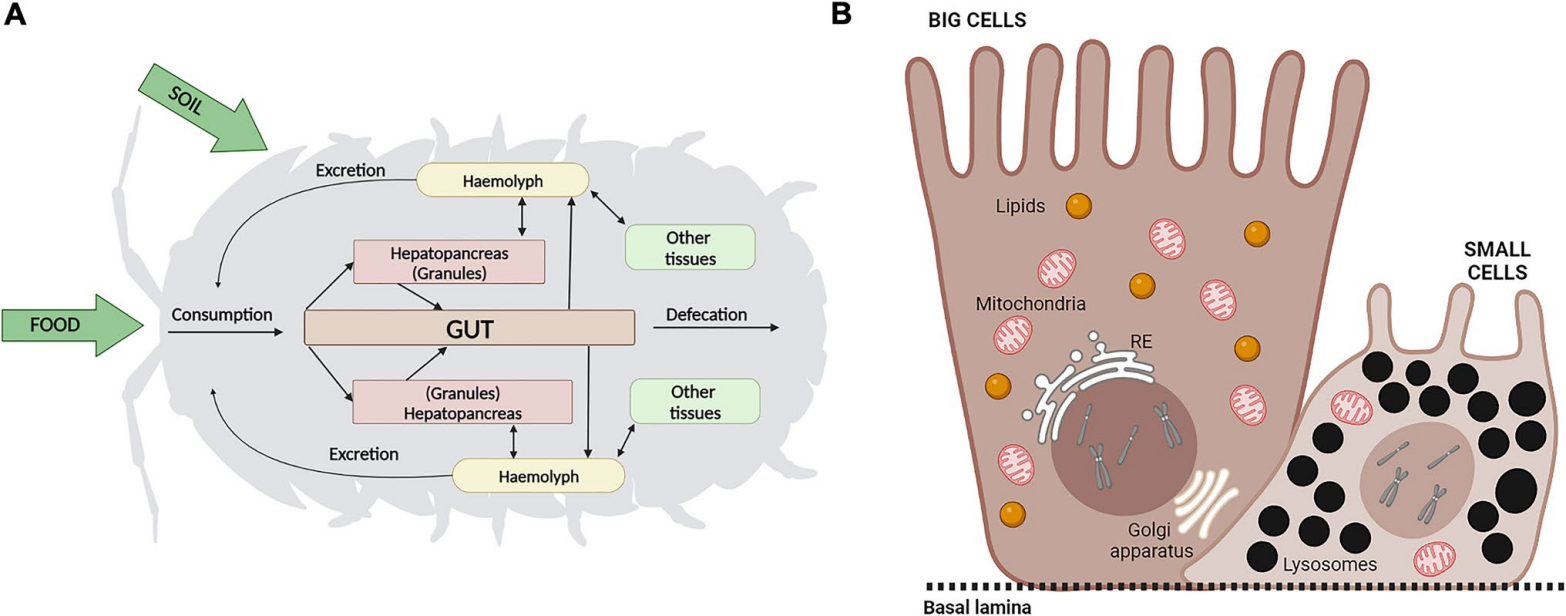
**A** Schematic diagram of the uptake and processing pathways of chemical pollutants in isopods. **B** Schematic diagram of structural and functional differences in S and B cells

Previous research (Donker et al. 1966; van Gestel et al. 2018) has evaluated metal uptake and excretion kinetics, taking in consideration both soil and food as pollution sources. The possible internal distribution of contaminants in the body of the isopod was also considered in our study (Fig. 1A), particularly for the digestive system.

The hepatopancreas is part of the digestive system and was identified as the main target tissue for contaminant accumulation, for example, the accumulation of heavy metals (Cu, Cd and Zn) deriving from different sources (e.g. agriculture, industry) (Jelassi et al. 2020; Kampe and Schlechtriem 2016; Pastorino et al. 2021; van Gestel et al. 2018); although representing about 5% of the dry weight of the animal, may contain more than 75% of zinc, 95% of cadmium, 80% of lead and 85% of total copper (Hopkin and Martin 1982).

The hepatopancreas contains two cell types (Fig. 1B), the small (S cells) and the big cells (B cells), that differ in their excretion behaviour (Kampe and Schlechtriem 2016; Van Gestel et al. 2018). The S cells have the role of accumulating metals; the B cells are renewed frequently, therefore playing the main role in excretion, and contain large stores of glycogen and lipids, constituting the main energy reserve of isopods (Hopkin and Martin 1982; Prosi et al. 1983; van Gestel et al. 2018).

In this study, flow cytometry (FC) is applied as the main methodologic approach to investigate cell viability and cell functions of the hepatopancreatic tissue of isopod, collected from sites at different degrees of ecological disturbance.

The innovation of this research is the use of FC in environmental ecological approaches. In fact, FC is a rapid, quantitative, robust and multi-parametric analysis that allowed us to investigate the biological system and detect how a generic pollution impacts (not only that due to heavy metals), particularly in the hepatopancreas and consequently in its two constituent cell types.

Differences in functional parameters can reveal the presence of environmental stress and, if correctly interpreted, delineate the scenario of pollutant triggers to which isopods are subjected.

In fact, in both cell types (S and B) of the hepatopancreas of the isopod *Porcellio scaber* (H. R. Köhler et al. 1996), it was demonstrated in an in vitro setting that heavy metal exposure led to ultrastructural alterations, the degree of which was found to be dose-dependent. Low metal concentrations caused reactions of distinct organelles, while comparably higher concentrations resulted in pathological changes in the epithelium of the hepatopancreas.

Therefore, we started the study of hepatopancreatic cells using flow cytometry protocols developed in a previous in vitro experimental setting (Manti et al. 2013) and newly generated, to set up standardised procedures and labelling protocols in this in vivo context.

The current study’s main goal is optimising the hepatopancreatic tissue disaggregation to collect cellular samples representative of the organ status (physiological or pathological). Furthermore, flow cytometric labelling protocols are designed to analyse the viability and the reactions of distinct organelles in order to obtain data indicative of the level of pollution of the habitat of sampled isopods, representing specific ecosystems.

## Results

### Distribution of S and B cells

Our previous work (Manti et al. 2013) demonstrated that the hepatopancreas of the terrestrial isopod *A. vulgare* (Isopoda, Crustacea, Latreille 1804) plays an important role in the bioaccumulation of contaminants, through the evaluation of biological effect on fresh hepatopancreatic cells contaminated in an in vitro controlled system.

**Fig. 2:**
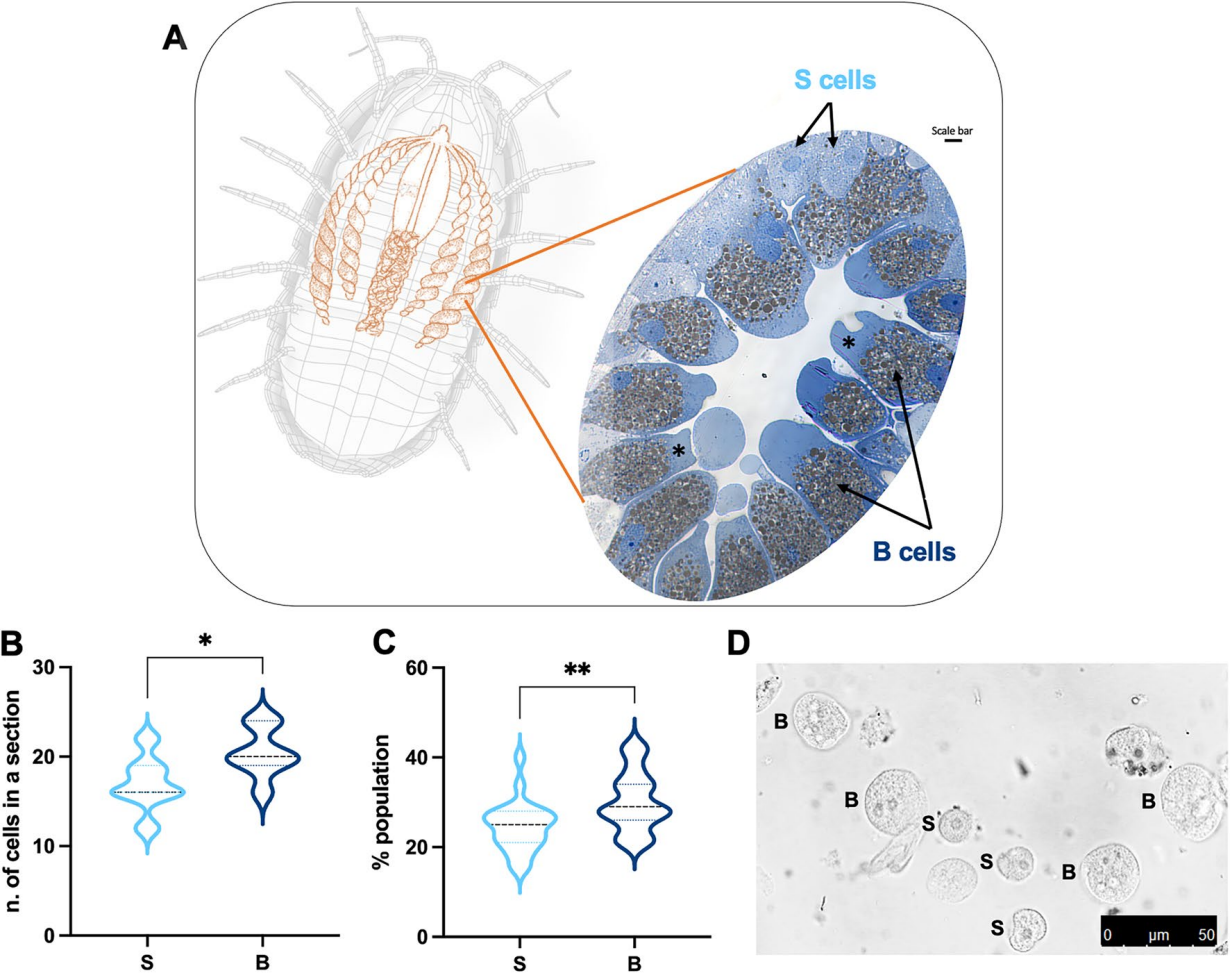
**A** Cell structure/morphology of the hepatopancreas, image taken by inverted microscope stained with toluidine blue: small cells in light blue, big cells in blue. Light micrograph of sections of a tubule where the apical cytoplasm of the B cells is engorged with large inclusions (arrows); note the B cells (asterisks) with disrupted apical membranes that are apparently undergoing apocrine secretion. Cell count by microscopic (**B**) and cytometric (**C**) analysis. *t*-Test with Mann–Whitney test revealing statistical significance: * = *p* < 0.05, ** = *p* < 0.01. **D** Representative bright-field image, obtained with a confocal microscope, of S cells and B cells after mechanical disaggregation

The presence of two different cell types (herein referred to as ‘small’ (S) cells and ‘big’ (B) cells) was detected for the first time through by flow cytometry by our group (Manti et al. 2013) and herein confirmed, in animals sampled from different sites, in an ex vivo uncontrolled system (Fig. 2A).

Cell counting, carried out by cytometric and microscopic analysis (Fig. 2B, C), showed a percentage of B cells slightly higher than S cells.

S and B cells forming the epithelium in the hepatopancreas differ in size (Fig. 2A, D). These subpopulations were evaluated after both disaggregation techniques, revealing slightly different percentages. In particular, B cells are counted as about 35–40% of the total cell suspension, while S cells amounted to about 60–65%, through manual disaggregation, whereas after automated disaggregation, the two cell types highlight more similar percentages: about 45% (S cells) and 55% (B cells). The latter cytometric quantitative data are also confirmed by microscopic observations (Fig. 2A, B, D).

### Selection of the more suitable disaggregation technique

The two disaggregation types were further evaluated. Briefly, the manual procedure (applied in a previous work (Manti et al. 2013)) and the automated one, innovative and more standardised (Montanari et al. 2022), were compared.

**Fig. 3:**
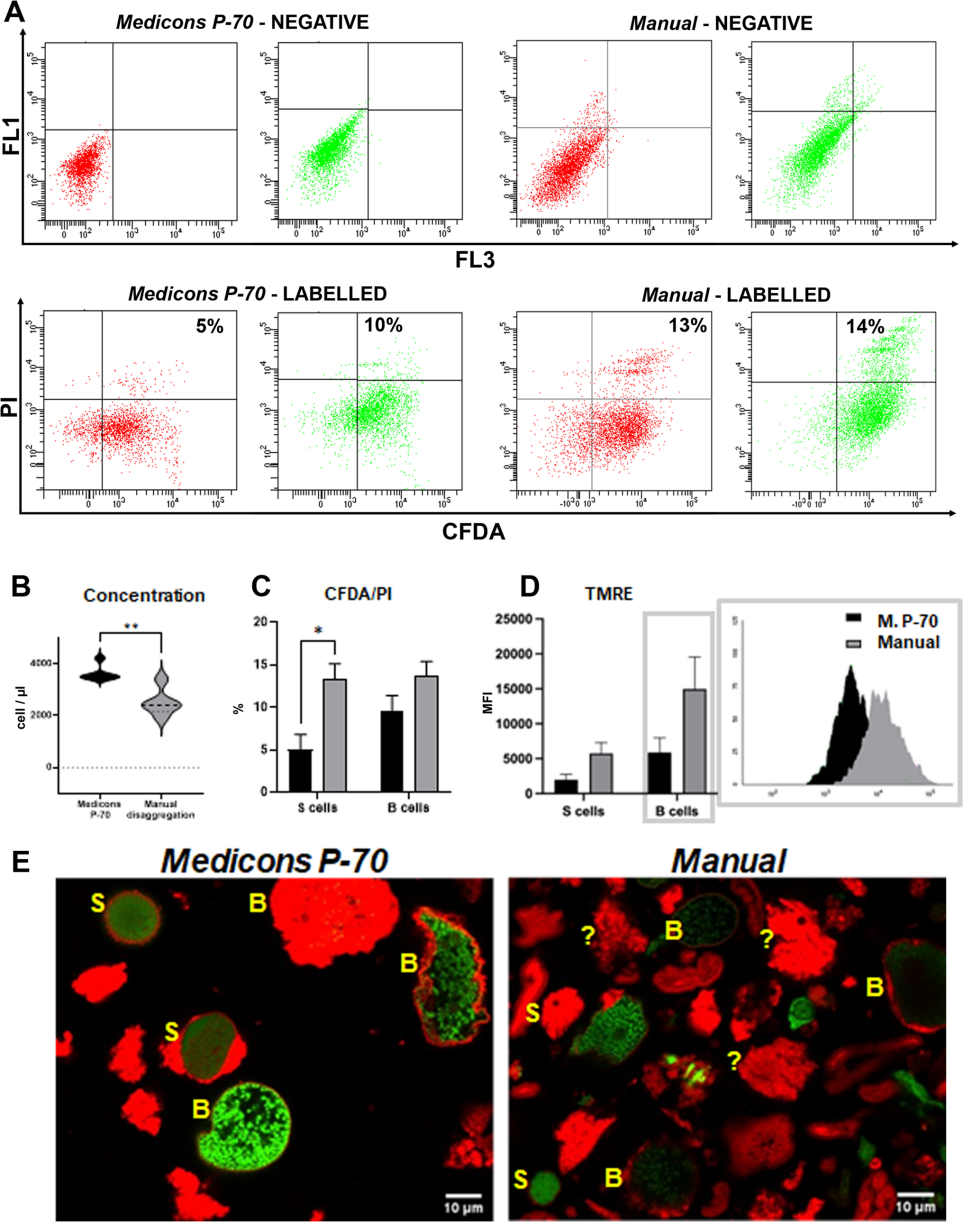
**A** Cytometric dot plot for the evaluation of CFDA vs propidium iodide (PI) for the different disaggregation procedures (Medicons P-70, manual disaggregation). B cells in green, S cells in red. **B** Cell concentration in differently disaggregated samples. *t*-Test with Mann–Whitney test revealing statistical significance: ** = *p* < 0.01; **C** histogram of the percentage of PI-positive cells between S and B cells obtained from different disaggregation. Two-way ANOVA with Bonferroni’s multiple comparison revealing statistical significance: * = *p* < 0.05; **D** histogram of TMRE fluorescence in S and B cells obtained from different disaggregation and cytometric histogram of TMRE MFI for B cells, which does not reveal a statistical significance. **E** Single confocal optical sections of nucleus (SYBR Green) and lysosomes (LTDR) of cells obtained from different disaggregation techniques. S and B capital letters indicate S or B cells, whereas question marks highlight cell fragments impossible to be identified

We applied the labelling CFDA vs propidium iodide for both assessing cell viability and excluding debris, detecting a greater PI positive percentage (dead/damaged cells) in manual disaggregation procedures whereas, after the mechanical-automated protocol (MP-70), PI positive events were present in lower percentages (Fig. 3A, B). All measurements were conducted on hepatopancreas cells of the same *A. vulgare* specimens, sampled from the same site. Therefore, any difference, even if slight, can be attributed to the different methods of disaggregation.

A high mitochondrial potential was registered, by means of TMRE dye (Fig. 3C, D), in the automatically disaggregated B cells. In Fig. 3D, there are histograms related to TMRE MFI (mean fluorescence intensities) indicate as follows: in violet, the mitochondrial membrane potential of cells from automated disintegration/Medicons P-70 and, in pink, the mitochondrial membrane potential of cells from manual disintegration.

The images deriving from confocal microscopy analysis (Fig. 3E) highlight elevated levels of cell damage in the samples obtained by manual method, while the use of Medicons disaggregated the tissue at best, allowing detachment of a great amount of well-preserved S and B cells (Fig. 3E). The cells in Fig. 3E are labelled with SYBR Green (green nuclei) and LTDR (red lysosomes). Data indicated that the best disaggregation procedure is performed by MediMachine II, not only for the membrane damage (underlined by PI positivity) that S and B cells are easily recognisable by FC.

### Mandatory tests: individuation of dead/damaged cells with enzymatic activity

Hepatopancreatic cells from individuals collected in different sites at different levels of ecological disturbance after tissue disaggregation were analysed by a single-blind control procedure (i.e. without informing the biologic researchers on the classification of the different sampling sites).

**Fig. 4:**
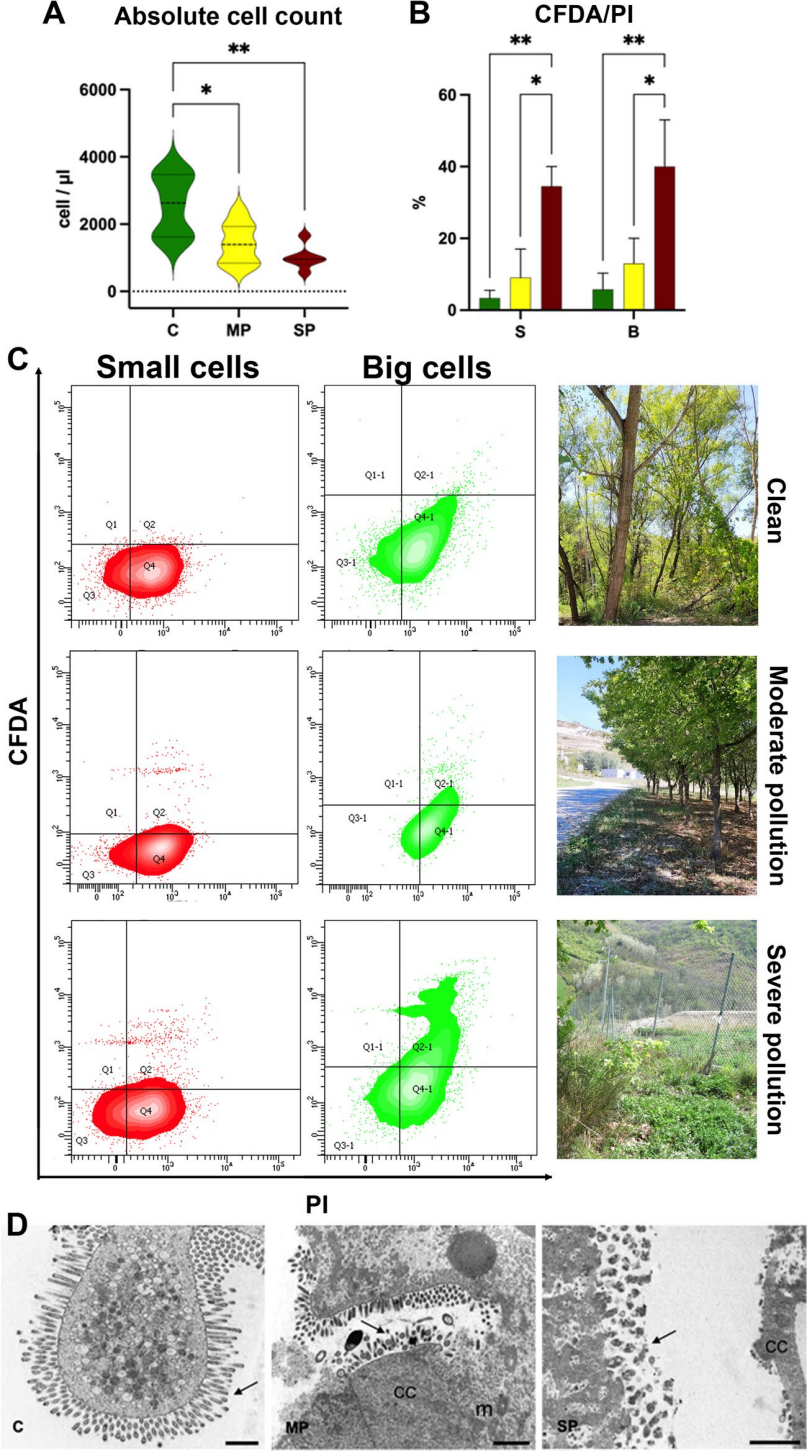
**A** Cell concentration/organ weight of hepatopancreatic cells from the different sites (C, clean; MP, moderate polluted; SP, severe polluted). *t*-Test with Mann–Whitney test revealing statistical significance: * = *p* < 0.05, ** = *p* < 0.01. **B** Histograms of the percentage of propidium iodide positive S and B cells of individuals from the different sites near the landfill sampled in the 2 years of sampling (C, MP, SP). Two-way ANOVA with Bonferroni’s multiple comparison revealed statistical significance: * = *p* < 0.05, ** = *p* < 0.01. **C** Contour plot of PI vs CFDA for C, MP and SP. In red S cells, in green B cells. Quadrants are placed on the basis of each specific autofluorescence value of the corresponding unlabelled sample. **D** TEM images for C, MP and SP samples. The progressive microvilli damage is evident. C and MP, bar 1 µm; SP, bar 2 µm. It is possible to observe: microvillus border disruption (arrow), condensation of some cytoplasm areas (CC) and mitochondrial alterations (m)

The main data from the mandatory tests is shown in Fig. 4. Cell counts of different samples highlight the highest number of hepatopancreatic cells in animals from potentially clean sites (C), whereas the overall counts decrease in moderate and severe pollution samples (MP and SP) (Fig. 4A). Cell concentrations (cells/µL) were calculated taking in consideration the starting organ weight (Fig. 4A).

Striking differences in PI-positive events were detected in samples of animals from different sites, contemporary demonstrating a different cellular response of S and B cells. Of note, both S and B cells show a higher dead/damaged cell rate in samples from isopods collected in SP sites, revealing an increase in CFDA/PI + events, compared to C samples (Fig. 4B, C). In Fig. Supplementary 1, the autofluorescence of the samples (C, MP and SP) can be observed.

This test can be considered mandatory for assessing the condition of hepatopancreatic cells, both S and B types, due to its ability to clearly demonstrate that percentages of damaged/dead cells increase with an increasing degree of pollution.

To confirm such main findings, samples were also analysed by transmission electron microscopy (TEM). Figure 4D shows a peculiar microvilli reorganisation from C to MP and SP samples. Cell surfaces are highlighted as being covered with microvilli of altered shape (MP and SP) or with spherical-shaped bacteria. We also observed changes in shape, size and density of microvilli. In some regions, microvilli were almost absent (SP). Similar observations resulted from light microscopy (Fig. 4D). In some cells, microvilli still retained their usual appearance, while in other cells they were short, sparsely distributed or even absent. Moderate and severe polluted samples showed several changes, including length and number decrease of microvilli. These tissue alterations clearly explain how stressed hepatopancreatic cells can be intercepted by the rapid, flow cytometric CFDA/PI test as PI-positive events, progressively increasing in highly polluted conditions.

### Ancillary tests

In order to bring out differences between C and MP samples and to attempt the impervious route of distinguishing different types of soil pollution (insecticides and pesticides, industrial waste, heavy metals, waste disposal, agricultural activities, oil spills and so on), other tests were set up.

By means of the SYBR Green dye, it is possible to distinguish further subpopulations in both S and B cells:

- Events with a very high fluorescence signal (SYBR Green I bright)
- Events showing a lower fluorescence signal (SYBR Green I dim)

These staining differences were considered in a previous publication (Manti et al. 2013) and are probably derived from both polyploid cells and from a major content of nucleic acids (DNA and RNA), associated with the presence of more than one nucleus or increased cell activity, respectively.

**Fig. 5:**
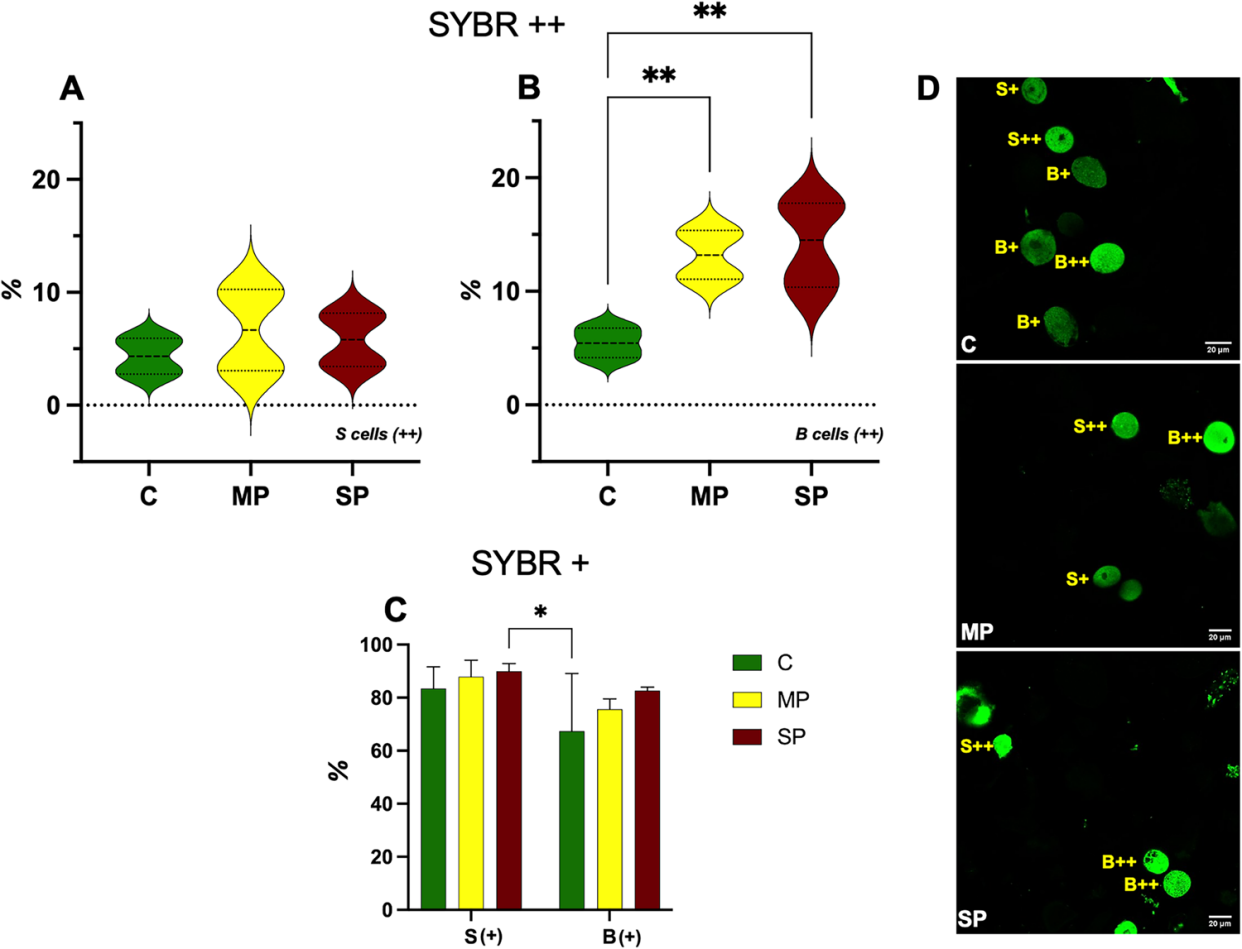
Histograms of the percentage of SYBR Green cells bright positive (+ +), S cells (**A**) and B cells (**B**), and dim positive cells ( +) (**C**) of individuals from the different sites near the landfill sampled in the 2 years (C, clean; MP, moderate polluted; SP, severe polluted). Ordinary one-way ANOVA (**A**, **B**) and two-way ANOVA (**C**) with Bonferroni’s multiple comparison revealed statistical significance: * = *p* < 0.05, ** = *p* < 0.01. **D** Single confocal optical sections of the nucleus (SYBR Green) of cells obtained from different samples (from C, MP, SP sites) bar 50 µm. In yellow, we highlight the presence of S or B cells

First, we assessed the two distinct SYBR Green I subpopulations in total cells of the samples, collecting the data depicted in Fig. 5, showing the highest percentage of SYBR Green bright cells in MP (S cells) and SP (B cells) samples (Fig. 5A, B), and the lowest percentages of SYBR Green dim events in C samples (Fig. 5C).

Of note, the percentage of B SYBR +  + (bright) cells appears to be directly proportional to the degree of pollution; differences in C, MP and SP classes are relevant and statistically significant (Fig. 5B).

The increase in SYBR Green fluorescence from clean to potentially polluted samples (SP) is also confirmed by confocal microscopy analysis of the same disaggregated cellular preparations (Fig. 5D).

### Assessment of impairment of cellular functions (mitochondrial potential and heavy metal accumulation)

To connect data on viability and subcellular organelles of disaggregated hepatopancreatic cell preparations, staining protocols adopted in research and clinical panels (Dong et al. 2019; Guha et al. 2019; Metryka et al. 2020; Scherr et al. 2018) were applied.

**Fig. 6:**
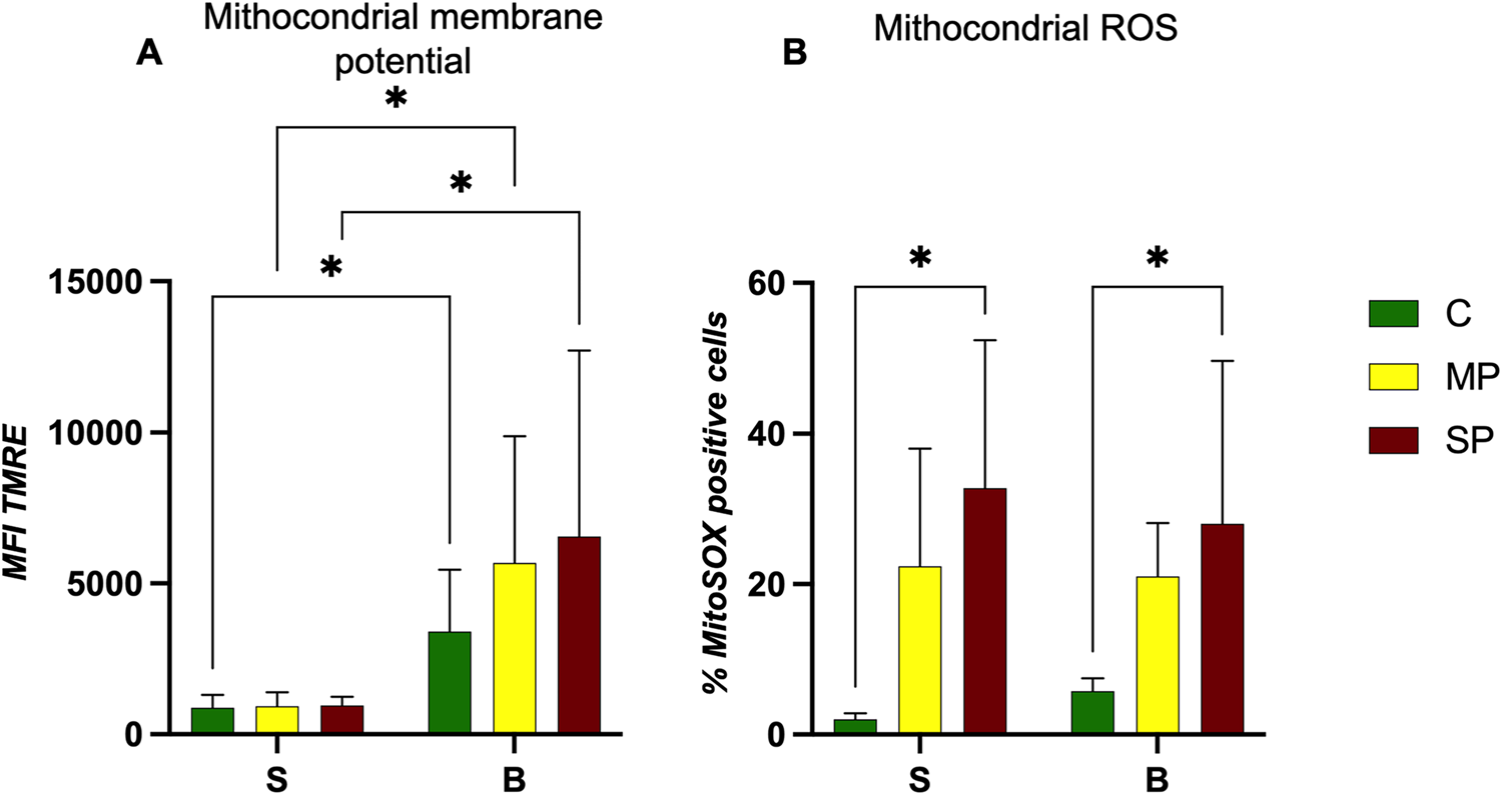
Histograms of mitochondrial membrane potential using TMRE dye (**A**) and percentage of mitochondrial reactive oxygen species using MitoSOX dye (**B**) in cells (small and big) from the different sites near the landfill (C, clean; MP, moderate polluted; SP, severe polluted). Two-way ANOVA analysis with Bonferroni’s multiple comparison revealed: * = *p* < 0.05

In particular, the mitochondrial network was studied by means of TMRE and MitoSOX probes (Fig. 6).

An increase in TMRE fluorescence was registered for B cells, although not significant: these data are in agreement with other researchers (Ghemari et al. 2021), finding an increase in mitochondria with increasing trace elements (cadmium (Cd) and zinc (Zn)), in an in vitro experimental setting, studied by atomic absorption spectrometry (AAS). Indeed, our results show that flow cytometric evaluation of mitochondria membrane potential by TMRE correctly underlines the relevant physiologic differences between S and B cells, in agreement with previous findings with other, not quantitative, time-consuming techniques (Štrus et al. 2019) reporting abundant mitochondria in the cytoplasm of hepatopancreatic B cells.

In order to better analyse mitochondrial activities, we performed a labelling by MitoSOX, able to trace mitochondrial ROS (mROS). Figure 6B highlights frequencies of cells actively producing mitochondrial ROS, with significant differences between C and SP sites, demonstrating an increase of mROS in response to progressive high pollution, in both S and B cells.

**Fig. 7:**
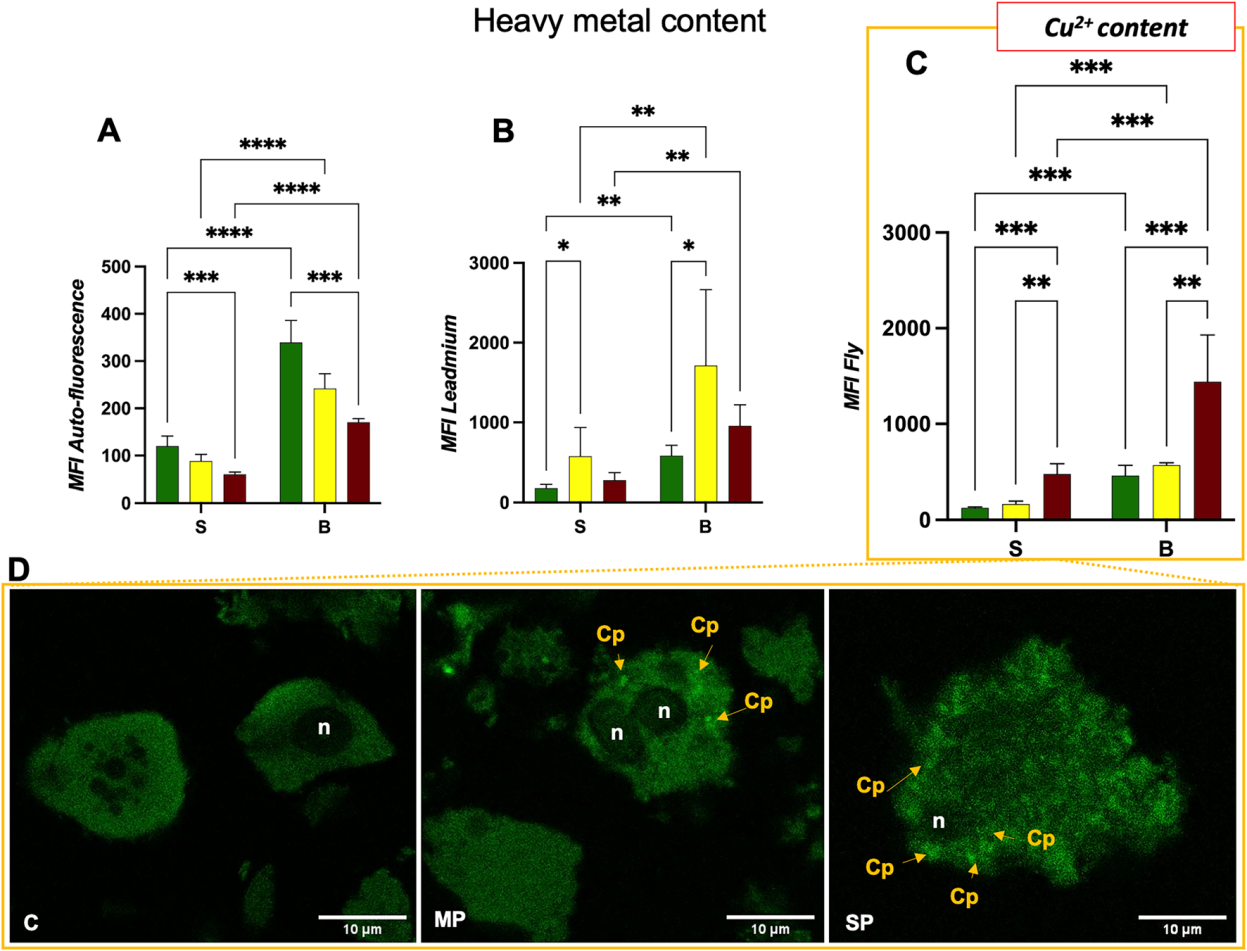
MFI histograms of autofluorescence (**A**), Leadmium (**B**) and Fly (the latter was developed by the research group from Urbino) (**C**) (without autofluorescence, AF) in cells from the different sites near the landfill (C, clean; MP, moderate polluted; SP, severe polluted). Two-ANOVA analysis with Bonferroni’s multiple comparison revealed: * = *p* < 0.05, ** = *p* < 0.01, *** = *p* < 0.001, **** = *p* < 0.0001. **D** Single confocal optical sections of cells from different sites (C, MP, SP) highlighted structures brilliantly labelled by the Fly probe, suggesting the presence of cuprosomes-like organelles (Cp) (n = nucleus), scale bar 10 µm

Autofluorescence, the natural emission of light by biological structures, may possibly hamper fluorescence-based techniques if not properly addressed and corrected (Larsen et al. 2021). Several endogenous fluorophores are known to cause autofluorescence in many tissues, for these reasons we evaluated autofluorescence in each instrument channel, with particular attention to FL1 (520 nm), corresponding to the wavelength of emission of several probes employed in the study (CFDA, SYBR Green, Leadmium, Fly). Indeed for hepatopancreatic cells, overall autofluorescence emission contains considerable information because it is the sum of autofluorescence contributions from fluorochromes involved in metabolism (i.e. NAD(P)H, flavins, lipofuscins, retinoids, porphyrins, bilirubin and lipids) (Montanari et al. 2022). Accordingly, samples from C sites show higher autofluorescence values compared to MP and SP samples (Fig. 7A), confirming that increasing pollution can decrease the enzymatic pool of a healthy organ.

The Leadmium dye was employed to monitor the possible accumulation of heavy metals. It is advertised as a detector of free intracellular cadmium (Cd^2+^) and lead (Pb^2+^). Of note, Leadmium MFI increased from C to MP sites but in SP sites it did not show the expected increase, highlighting values similar to that of C samples. Although this finding will be further studied, it could depend on the high mortality rate registered in the SP samples (as clearly shown in Fig. 4C, D about CFDA/PI). In fact, cells with compromised viability lost their main functions, including the ability to accumulate heavy metals.

Indeed, for the detection of divalent metals, the Fly probe was employed: this is a yet uncommercialised fluorophore, developed by a research group from Urbino (Ambrosi et al. 2009; Canonico et al. 2022), which provides the main indications on the presence of copper (Cu^2+^): it was employed to detect intracellular Cu(II) storage and its possible content changes. This dye emits green fluorescence, if bound to Cu(II) in a low polar environment (Amatori et al. 2012; Canonico et al. 2022), as intracellular membranes, suggesting its use as Cu(II) probe. Fly biomarker suggests a statistically significant and proportional increase in fluorescence between samples C, MP and SP; B cells showed greater fluorescence than S cells (Fig. 7C). In addition, high significance was found between S and B cells at all sites sampled. Indeed, confocal images paired to flow cytometric quantitative data show that in unpolluted hepatopancreatic cells a dim, diffuse green fluorescence appears. The B cells, larger and protruding into the lumen of the hepatopancreatic tube, in the animal digestive system, are shown in Fig. 7D. It is possible to observe centrally, one or two irregularly shaped nucleus sections (n). Punctated, bright green fluorescence appears in MP and SP samples and these organelle-like structures (Cp) are distributed throughout the cell.

As several studies have shown, isopod efficiently sequesters copper in ‘cuprosomes’ within hepatopancreatic cells (Hong-Hermesdorf et al. 2014; Kille et al. 2015; H.-R. Köhler 2002; Schultz et al. 2015); others have shown that X-ray spectra of membrane-limited vesicles, called cuprosomes, reveal a main accumulation of copper, although amounts of other metals (Prosi et al. 1983) can be found.

Although further analyses are mandatory to characterise such structures, our results would confirm the presence of ‘cuprosomes’ in *A. vulgare*.

## Materials and methods

### Ethical statement

The present study was performed on public lands, if in private land with the landowner’s approval. No specific permissions were required to conduct the present experiments at these locations. Our study did not involve endangered or protected species. Ethical approval was not needed for using *Armadillidium vulgare* as a study system in ecological studies. All experimental procedures and animal manipulations did not require an ethics statement.

Animal welfare and the relevant experiment were carried out in compliance with the guide for the care and use of laboratory animals. Indeed, organisms with abnormalities, moulting animals and pregnant females were excluded, as indicated by other researchers (Morgado et al. 2022).

### Experimental design

Our study develops in two parts: the ecological-environmental part and the biological one. Here these steps are summarised:

1. Appropriate sites for isopod sampling (e.g. potentially polluted and more natural areas) were identified.
2. Sample collection was carried out.
3. Preparation of the isopod samples for biological hepatopancreatic analysis in a work setting to identify the most effective disaggregation technique.
4. Application of FC, as the main analytic approach.
5. Data analysis and interpretation on acquired samples were carried out.

To improve the data’s robustness, this study belongs to a sampling plan divided into two different years. In both years, the analyses were carried out in vivo.

### Method’s assessment for the ecological-environmental part

In the ecological-environmental part, sites with different degrees of ecological-environmental disturbance were identified and sampled.

The sites selected for the sampling were empirically classified according to the level of disturbance and naturalness, based on the location of the potential stress source and of the well-known ecological-environmental characteristics of the sites, without informing the cytometric lab on this pre-classification:

- Clean (C): potentially not polluted and/or stressed sites (e.g. forest)
- Moderate polluted (MP): potentially moderately polluted and/or stressed sites (e.g. hedges near roads)
- Severe polluted (SP): potentially polluted and/or stressed sites (e.g. industrial areas)

The first phase was to develop a sampling plan and work protocol inspired by previous studies, where samples were collected at variable distances from the stress source (Mazzeo et al. 2013; Nannoni et al. 2015, 2017).

After we identified the macro sampling area (e.g. landfill), we studied more in detail the territory in which it was located.

The study of the area was done using thematic cartography (e.g. land use, aerial photos) through Quantum GIS software (QGIS, Open Source Geographic Information System) and programs such as Google Earth and various types of documentation (e.g. management plans).

**Fig. 8:**
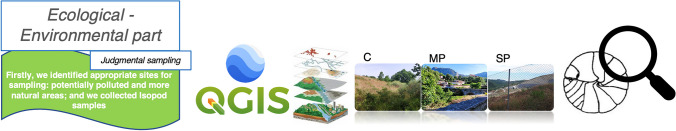
Step-by-step procedures for the ecological part. Study of the area and identification of sampling sites with different levels of disturbance: C (clean), MP (moderate polluted), SP (severe polluted). Terrestrial isopod sampling

This study of the area and the knowledge gained about the species *A. vulgare*, such as habitat and habits, allowed us to select potentially appropriate sites for the presence of the species (Fig. 8), and also to classify them empirically (C, MP, SP) based on the observed environmental ecological characteristics. Therefore, allowed us to proceed with judgmental sampling, considering that it potentially requires fewer samples than other sampling schemes (e.g. systematic or random).

Preliminary field inspections were conducted to verify if our assumptions were true and, therefore, if the species was present at the stations identified by the mapping. Sampling was carried out from April to September. Isopod picking was carried out in the cooler hours (early morning or evening) when these animals are most active and, therefore, more readily found.

Species were identified using dichotomous keys and scientific articles (Noël and Séchet 2007; Taiti and Judson Wynne 2015; Vandel 1960, 1962).

#### Study area and sampling strategy

Samples were collected at the Ginestreto landfill, and in its proximity, in the municipality of Sogliano al Rubicone (FC, North Italy). In total, more than 10 sites were sampled over the two survey years, sampling from April to September. Approximately 7 isopods were sampled at each site, with a total amount of almost 100 isopods sampled over the 2 years near the landfill site.

The sites for the sampling were located within the landfill (*n* = 3), within the landfill boundary (*n* = 3) and along the SW-NE transect drawn (*n* = 4) (Fig. Supplementary 2).

As can be seen from Fig. Supplementary 2, there are several stations inspected in the study area where the presence of the species was found, only in a few of which enough individuals were collected for biological analysis.

At each site, individuals of *A. vulgare* were sampled within a circular area (radius 5 m), inspecting the various microhabitats in each station.

Sampling isopods required the use of a hand-rake, gloves and a container to correctly store viable individuals for analysis.

Catches occurred in the spring and summer months in the cooler hours, sometimes during nocturnal hours.

### Method’s assessment for the biologic part

Flow cytometric and microscopic analyses were conducted on hepatopancreatic cells of terrestrial isopods to search potential stress markers/indicators induced by critical environmental conditions.

In the starting phase of the study, to collect hepatopancreatic cells, we applied two different mechanical disaggregations: manual and automated (MediMachine II) tissue disaggregation (Montanari et al. 2022). The comparison was performed on samples from the same sites.

Specific cell function probes, already emerged in a previous in vitro work (Manti et al. 2013), were utilised, revealing a striking ability to detect cell damage in the hepatocytes and, contemporary, new parameters were specifically selected to highlight several subcellular alterations in *A. vulgare*.

Cell death, ROS content, DNA content, mitochondria and lysosome network were evaluated to identify the stress to which cells are subjected. Dyes are indicated in the next session. Analyses were carried out mainly by FC and confocal laser scanning microscopy (CLSM).

#### Tissue disaggregation and final preparation of the representative samples

**Fig. 9:**
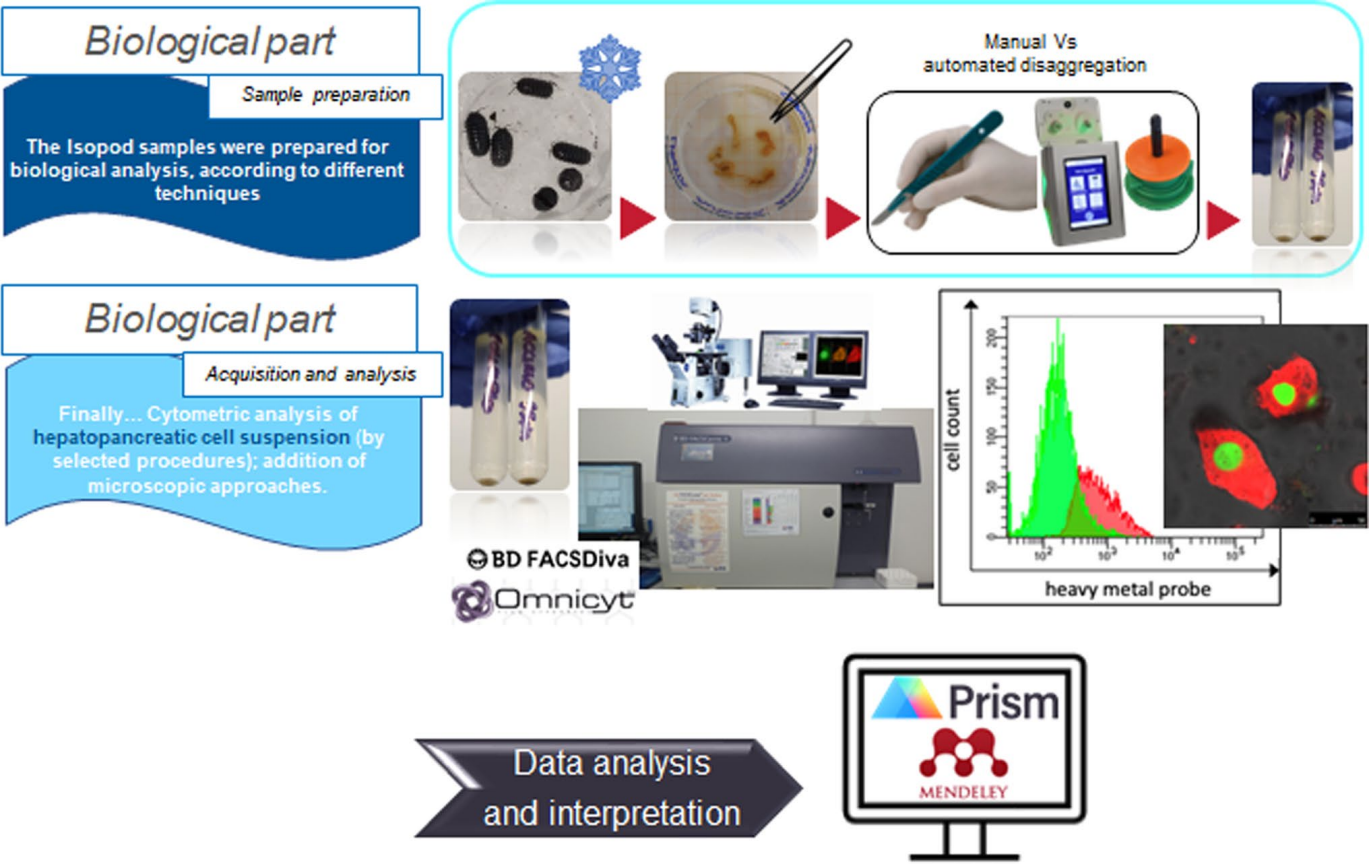
Step-by-step procedures on the biological part. Sample preparation consists of putting the isopods in a Petri dish on ice to reduce motor activity. The second step is the extraction of the hepatopancreas. This is disaggregated (automatically or manually) and centrifuged to obtain a cell pellet for analysis. Finally, the sample is acquired and analysed using a cytometer. Sometimes these cytometric analyses are supported by microscopic analyses. This is followed by data analysis and interpretation of the results

A sample of 5–7 animals was considered representative of the site (Fig. 9). After dissection by tweezers, isolated hepatopancreatic tubes were put in a Petri dish and resuspended in PBS + 5% FBS-EDTA, on ice to keep the sample well preserved. Then, in selecting the best disaggregation practice, hepatopancreas (from sample of 5–7 animals) were managed by two comparative protocols: (1) gently crumbled with a scalpel by pressing them against the Petri dish, (2) by automated-mechanical procedure performed with MediMachine II (CTSV s.r.l.). In brief, disposable Medicons—P-70 µm were filled with 1 mL of PBS, before placing the hepatopancreas tubules. Tissue fragments were dissociated 2 times for 30 s at a constant speed of 100 rpm and a final washing step for 10 s was performed. Cells obtained by both mechanical were filtered with 70-µm mesh filter (Filcon; CTSV s.r.l., Bruino, Turin, Italy), resuspended in 500 µL PBS and centrifuged for 10 min at 467 × g at 4 °C.

#### Sample labelling and flow cytometry

Three different fluorescent labelling were employed, according to previous work (Manti et al. 2013). Briefly, 1 mg/mL acetone stock solution of fluorescein diacetate (CFDA; Molecular Probes, Eugene, OR, USA), 1 mg/mL stock solution of propidium iodide (PI; Sigma-Aldrich, St. Louis, MO, USA) and SYBR Green I (Molecular Probes, Eugene, OR, USA) used at a final concentration of 1:10,000 were added to the different cell suspensions. For mitochondrial features, tetramethylrhodamine ethyl ester perchlorate (TMRE; Sigma-Aldrich, St. Louis, Missouri, USA) and MitoSOX Red superoxide indicators (Thermo Fisher Scientific, Waltham, MA, USA) were used. TMRE is a mitochondrial membrane potential (MMP) specific stain able to selectively enter the mitochondria depending on MMP, producing a red fluorescence. TMRE 40 nM was added to the samples 15 min before the acquisition time. MitoSOX Red is a fluorogenic dye specifically targeted to mitochondria in live cells. Oxidation of this probe by the superoxide that is contained in the mitochondria produces a red fluorescence. Cells were stained with 5 μM MitoSOX Red in PBS for 10 min.

Heavy metals are ubiquitous environmental contaminants with widespread toxicity; we used Leadmium™ Green AM (Thermo Fisher Scientific, Waltham, MA, USA) to detect intracellular lead and cadmium. We added 4 µL of Leadmium™ Green AM dye working solution (prepared from Leadmium™ Green AM stock solution in 1:10 saline) to all tubes and incubated them at room temperature for 30 min, protected from light.

For Cu(II) detection a new, not commercially available probe, named Fly, was used, synthesised as reported by Ambrosi et al. (2009). Fly probe was used at a concentration of 10 µM, following 10-min incubation in the dark (Canonico et al. 2022).

Each dye according to the information provided is part of mandatory (generic biomarkers) or secondary (specific biomarkers) analyses (Table 1).

**Table 1:** Dyes used for analysis and respective information provided. Each dye shows the test type in which it was placed

*Dye*	*Information provided*	*Type of test*
CFDA/PI	Enzymatic activity and cell-membrane integrity/cell dead	Mandatory
SYBR Green I	Double-stranded DNA (dsDNA)	Ancillary
TMRE	Changes in membrane potential in mitochondria	Assessment of impairment of cellular functions
MitoSOX	Mitochondrial ROS in live cells	Assessment of impairment of cellular functions
Leadmium™ Green	Intracellular lead and cadmium levels with high sensitivity	Assessment of impairment of cellular functions
Fly	Cu(II) detection	Assessment of impairment of cellular functions

Samples were processed by flow cytometry, by means of a FACSCanto II (Becton Dickinson, Franklin Lakes, NJ, USA) instrument equipped with 405-nm, 488-nm and 633-nm lasers. Data were analysed using FACSDiva TM software (Becton Dickinson Biosciences). Absolute counting was performed by Omnicyt Flow Cytometer (Cytognos SL, Santa Marta de Tormes, Salamanca, Spain).

#### Sample labelling and confocal microscopy

We performed microscopic analysis to confirm the results obtained by flow cytometry. The images were acquired by a Leica TCS SP5 II confocal microscope (Leica Microsystem, Germany) with 488-, 543- and 633-nm illumination and oil-immersed objectives and averaged in real time using a line average to reduce random noise. The images were further processed and analysed in ImageJ software (National Institutes of Health, Bethesda, MD, USA).

#### Transmission electron microscopy (TEM)

Hepatopancreas tubules for TEM were dissected into smaller parts. The samples were fixed in 2.5% glutaraldehyde in 0.2 M phosphate buffer for 1 h, rinsed and then post-fixed in 1% OsO4 (phosphate-buffered) for 1 h at 4 °C. After samples were dehydrated using an ethanol series (50%, 70%, 80%, 90%, 95% and 100%) for 15 min each at RT and embedded in araldite. Polymerisation was conducted at 60 °C for 72 h. Specimens were either cut in series of semithin (1–2 μm) stained with 1% toluidine blue or ultrathin Sects. (70–80 nm) using diamond knives. Ultrathin sections were placed on grids then contrasted with Uranyless (45 min) and lead citrate (25 min). The sections were examined using a Philips 10 at 80 kV transmission electron microscope (Burattini et al. 2022).

#### Statistical analyses

Descriptive analyses were performed using media, and ± standard deviations (SD) were appropriated. The different sampling sites (clean (C), moderate polluted (MP) and severe polluted (SP)) were compared through analysis of variance (ANOVA) approaches. One-way ANOVA (represented by violin graphs) or two-way ANOVA (histogram graphs) were followed by a Bonferroni post hoc test, and *t*-test (violin graphs) with Mann–Whitney test. The *p* values less than 0.05 were considered statistically significant. Bonferroni’s multiple comparison test and Mann–Whitney test revealed statistically significant: * = *p* < 0.05, ** = *p* < 0.01, *** = *p* < 0.001, **** = *p* < 0.0001. All statistical analyses were performed using GraphPad Prism 9.0.0 (GraphPad software, San Diego, CA, USA). In particular, we performed three experiments to optimise the disaggregation method on isopod hepatopancreas, being this method yet validated by the same group (Montanari et al. 2022). For the diagnostic analyses, a sample of 5–7 animals was considered representative of the site. As previously stated, in total, more than 10 sites were sampled over the two survey years, with a total amount of almost 100 isopods sampled over the 2 years near the landfill site.

## Discussion

Although no standard test guidelines are available for assessing chemical toxicity to isopods, they are used as test organisms, applying different routes of exposure (food, soil), different test durations and different endpoints (van Gestel et al. 2018). Any change in the isopod population, diversity and life cycle can indicate relevant pollution levels (Sfenthourakis and Hornung 2018; van Gestel et al. 2018).

Previous studies have shown that the level of accumulation in the organism of isopods correlates with the level of contamination in rural, suburban and urban sites (Papp et al. 2019; Simon et al. 2016), and that the level of accumulation depends on the level present in food and soil (Heikens et al. 2001; Van Straalen et al. 2001). The accumulation pattern appears to be species-dependent (Pastorino et al. 2021): in our study we choose the species *Armadillidium vulgare* (Latreille, 1804) (Crustacea, Isopoda, Oniscidea), already studied in a previous in vitro model of artificial pollution.

The analysis of target tissues, as hepatopancreas, is another emerging approach (from a cytologic/histologic level) to detect contaminant accumulation from different sources and, particularly, cell damages caused by substances hardly identifiable or present only in traces, and synergically acting to decrease cell viability.

In this study, we investigated cell functions and viability of the hepatopancreatic cells (S and B cells) of isopods from sites of different degree of ecological disturbance, in order to detect possible differences in stress parameters and to verify if these parameters correspond to the environmental stresses. We successfully applied mechanical-automated procedures previously optimised on murine and rat tissues (Montanari et al. 2022).

The appropriately disaggregated cells were rapidly, efficiently and quantitatively analysed by multicolour FC (Auguste et al. 2020; Barmo et al. 2013; Canesi et al. 2008; Ciacci et al. 2011; Souza et al. 2023). This methodology that can be successfully applied to analyse hepatopancreatic cells from terrestrial isopods in order to conduct a so-called environmental diagnosis from a cytologic/histologic point of view, aimed to protect environmental welfare and human health (Abelouah et al. 2023; da Silva Souza et al. 2020; Plutino et al. 2022; Vieira et al. 2021). The current framework is partially derived from a previous work on an artificial pollution model (Manti et al. 2013), i.e. for CFDA/PI (viability) and SYBR Green I (DNA content) tests.

The cell viability parameter could be considered as the first test to define the state of the sample. This cell condition could be compromised by various cell death forms as necrosis (Gautam et al. 2020), apoptosis (Kumari et al. 2022), autophagy (Canonico et al. 2016) and pyroptosis (Bertheloot et al. 2021) due to possible pollutant accumulation and must be accurately quantified. The CFDA/PI assay, tracing cell viability, shows percentages of PI events significantly increased in progressively polluted sites, in both S and B cells. We used flow cytometry, transmission electron microscopy and confocal microscopy to describe and detect cell death, with a quantitative assessment of cells with depolarised mitochondria, helping to establish whether there is the relationship between cell death/damage and the impairment of mitochondria. TEM images ‘translate’ the flow cytometric findings in the direct visualisation of cell integrity and classical morphology of both hepatopancreatic cell types. The global predominant features were microvillus border disruption, condensation of some cytoplasm areas and mitochondrial alterations, in agreement with Jelassi and coworkers (Jelassi et al. 2019, 2020).

In addition, our study, coupled to the activation of cell death and the other measurements, aimed to highlight the mechanisms that take part in homeostasis loss or maintenance, as autofluorescence profiles, mitochondria status and mitochondrial ROS (Fan et al. 2021), the last identified by several researchers as the main actors of oxidative stress, descending from pollution (Kahremany et al. 2021; Sadiktsis et al. 2023). Regarding data collected by means of Leadmium, our results are in agreement with other studies (Rost-Roszkowska et al. 2020a, b; Rost-Roszkowska, Poprawa, Chajec, Chachulska-Żymełka, Wilczek, et al*.*, 2020), showing that short-term intoxication causes intensification of autophagy and digestion of reserve material, while long-term exposure to heavy metal causes activation of cell death processes. They conclude that short- and long-term exposure of soil centipede to cadmium affects different mechanisms and processes of cell death (Rost-Roszkowska et al. 2020a, b; Rost-Roszkowska, Poprawa, Chajec, Chachulska-Żymełka, Wilczek, et al*.*, 2020). Our results, showing an increase of intracellular cadmium content in MP samples but its decrease in SP samples, can correlate with the different findings for short-term and long-term exposure to cadmium, described by Rost-Roszkowska and coworkers (Rost-Roszkowska et al. 2020a, b; Rost-Roszkowska et al. 2020a). They reported that short-term exposure caused an increase in the cadmium concentration in the animal body, whereas after the long-term exposures the cadmium concentration in the organism of the animals decreased below the level described for the control group. Similarly, the decrease in Leadmium fluorescence (heavy metal concentration) in these animals is probably related to intense cell death (especially late apoptosis and necrosis), due to which damaged cells, as well as those having accumulated numerous toxic substances, are removed from the body and these evidences are traced by absolute cell counting and viability test. On the contrary, Fly staining shows a different trend. In biological systems, Cu is a crucial trace element of various important enzymes; it could undergo redox transitions between the Cu^2+^ and Cu^+^ state (Dan Zhao et al. 2019; Kehrer 2000; Thomas et al. 2009). This characteristic of Cu also makes it potentially harmful when Cu levels exceed the physiological needs (Bremner 1998; Dan Zhao et al. 2019; Kadiiska and Mason 2002; Seven et al. 2020). Cellular Cu homeostasis system tightly and effectively regulates the level of this metal by regulating the process of influx, distribution, sequestration and efflux of Cu (Balamurugan and Schaffner 2006; Culotta et al. 1999; Gaetke et al. 2014; Mercer and Llanos 2003; O’Halloran and Culotta 2000; Puig and Thiele 2002).

When excessive Cu overburden the Cu homeostasis maintaining in the organisms, it could cause oxidative stress through the induction of generation of reactive oxygen species (ROS), such as superoxide anion (O_2_^−^), hydrogen peroxide (H_2_O_2_) and hydroxyl radical (HO·) (Bremner 1998; Gaetke and Chow 2003; He et al. 2017; Kadiiska and Mason 2002; Kehrer 2000; Thomas et al. 2009).

The protocol we set up represents a method to highlight biological effects applicable as biomarkers for an assessment of the adverse impact of pollution (including heavy metals) exposure. To this regard, considering the importance to be informed on the internal concentrations of major pollutants in organisms, we are analysing selected sample by ESEM-EDX (environmental scanning electron microscope with an energy-dispersive X-ray spectrometer) to investigate the presence of specific heavy metal.

The analyses were conducted for the first time on isopods from sites at different conditions of ecological disturbance, through a cytometric re-interpretation of ecological-environmental parameters. Significant differences in cell functional parameters were found, highlighting that isopod hepatopancreatic cells can be efficiently analysed by FC and represent standardisable, early biologic indicators, tracing environmental-induced stress through cytologic/histologic analyses.

## Conclusion

In our research, we demonstrated the best tissue disaggregation technique (automated-mechanical procedure), which we successfully employed to analyse hepatopancreatic cells from terrestrial isopods in order to conduct a so-called environmental diagnosis from a cytologic/histologic point of view.

Indeed, we applied the flow cytometry technique to study the stress of *Armadillidium vulgare* hepatopancreatic cells. Isopod samples, taken from sites with varying degrees of ecological-environmental disturbance, show cell damage in proportion to the level of stress in which they live. These results set the basis for the development of an environmental quality index that considers the cellular state and uses a rapid, efficient and multi-parametric technique such as flow cytometry.

### Supplementary Information

Below is the link to the electronic supplementary material.

Supplementary file1 (PDF 352 KB)

## Data Availability

Data and relevant materials will be available from the corresponding author through email.
