# Supplementary material for: Flow cytometric analysis of hepatopancreatic cells from Armadillidium vulgare highlights terrestrial isopods as efficient environmental bioindicators in ex vivo settings
Source: Environ Sci Pollut Res Int. 2024 Jan 9;31(6):9745–63. doi: 10.1007/s11356-023-31375-x (PMC10824867; doi:10.1007/s11356-023-31375-x)
Supplement: Supplementary file 1 — Supplementary file1 (PDF 352 KB) [file 11356_2023_31375_MOESM1_ESM.pdf]

Supplementary

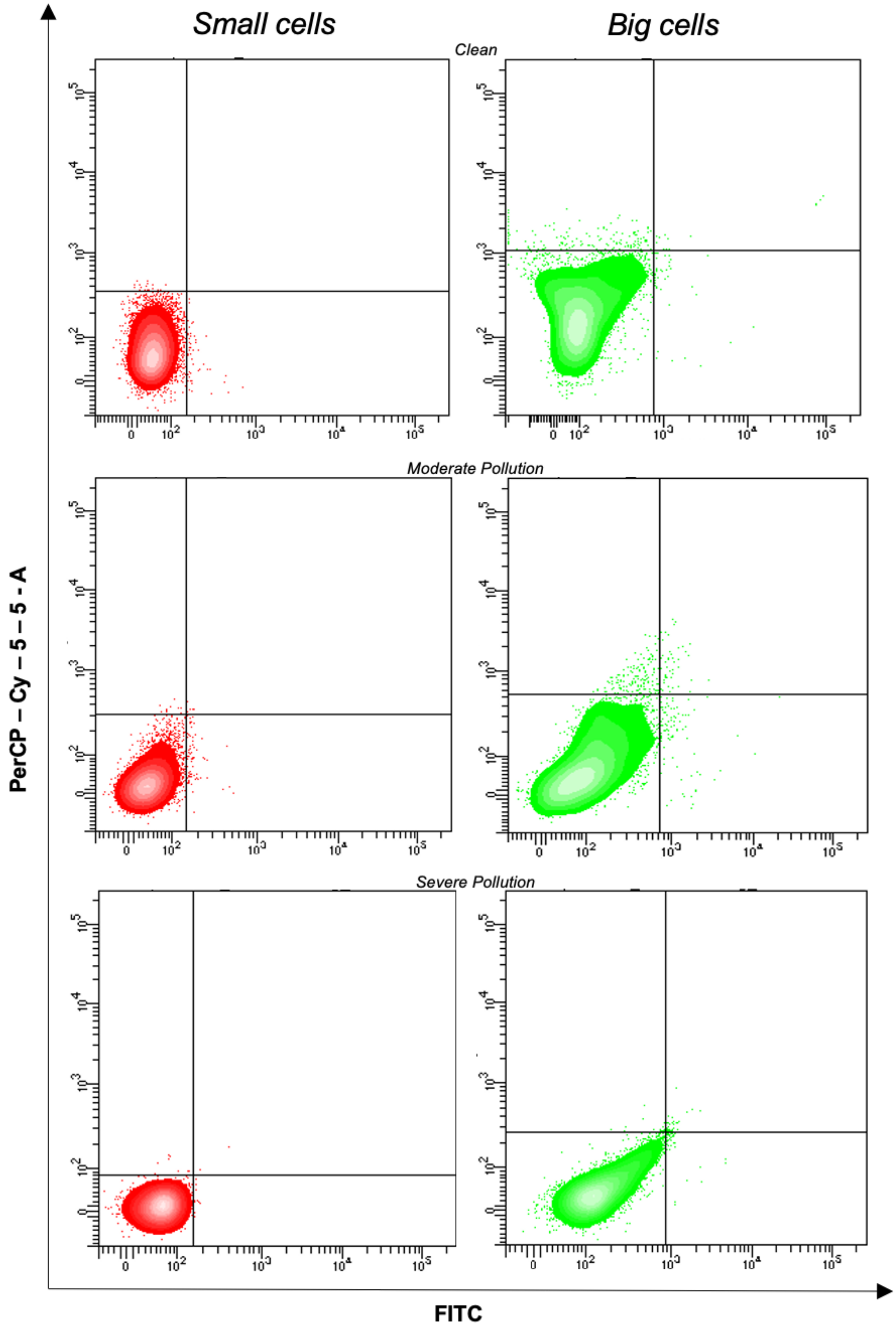

Fig Supplementary 1. S cells in red and B cells in green. Gating strategy about cytometric dot plot in Fig 4 for the evaluation of Propidium Iodide (PI) vs. CFDA for the three different sampling sites: C, MP, SP.

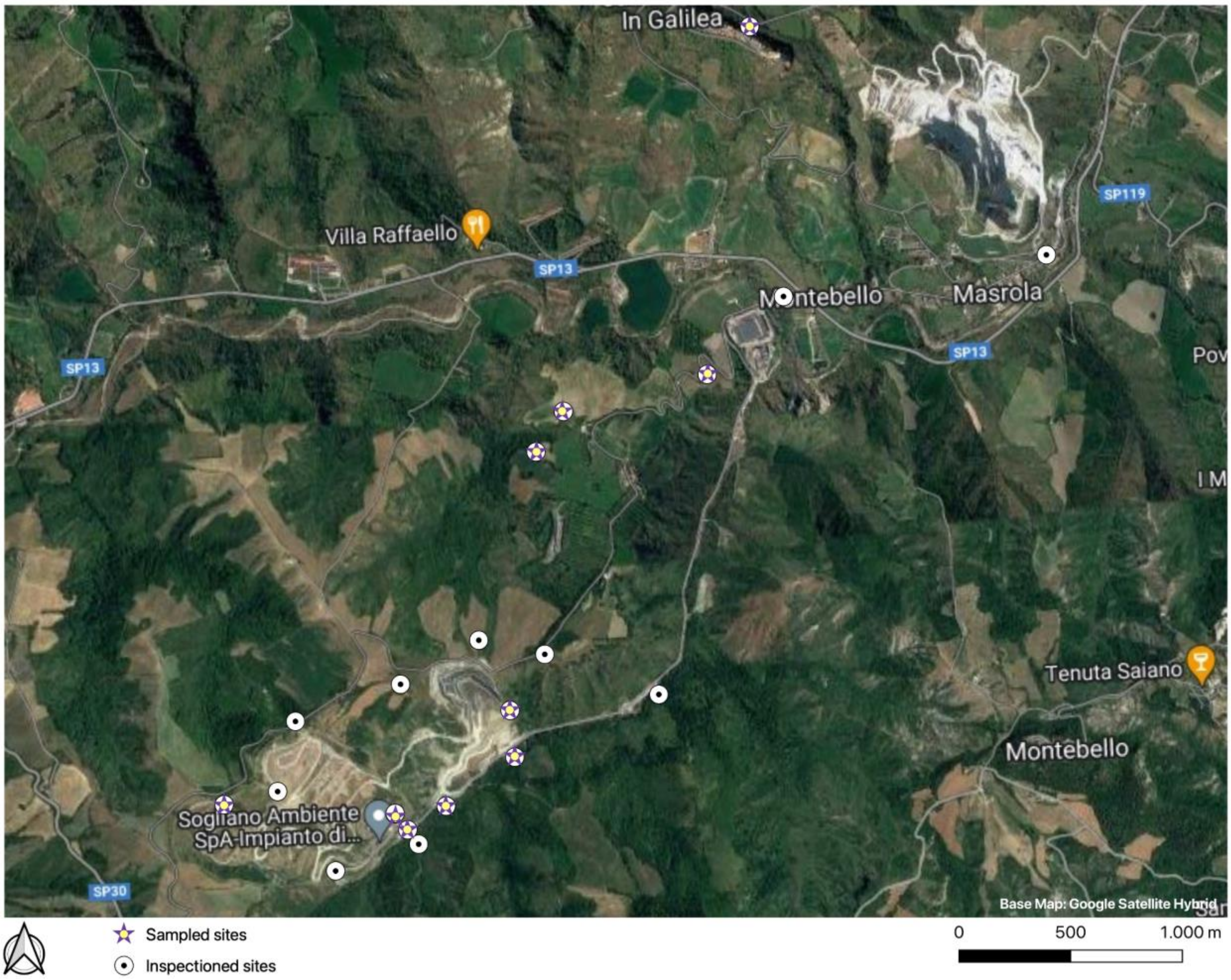

Fig Supplementary 2. Inspected sites where the presence of the species was found, and sampled sites where a good number of Isopods needed for analysis were found.
